# Wildlife Conservation in Tropical Savanna Ecosystems

**DOI:** 10.1155/2019/1573593

**Published:** 2019-03-14

**Authors:** Edson Gandiwa, Never Muboko, John F. Mupangwa

**Affiliations:** ^1^School of Wildlife, Ecology and Conservation, Chinhoyi University of Technology, Chinhoyi, Zimbabwe; ^2^Department of Animal Science, Faculty of Agriculture and Natural Resources, University of Namibia, Neudamm Campus, Windhoek, Namibia

The objective of this special issue is to address the diverse issues and developments related to wildlife conservation in tropical savanna ecosystems, given the challenges related to climate change, habitat fragmentation, illegal hunting and trade in wildlife products, and sustainable utilization. Many papers were submitted, and after a thorough peer-review process, six papers, i.e., five original research papers and one review paper, were selected to be included in this special issue. These research studies provide invaluable insights into understanding contemporary issues in wildlife conservation and scientific evidence for adaptive management and sustainable conservation.

The paper by V. K. Munyaka and E. Gandiwa presents an assessment of how the giraffe species (*Giraffa camelopardalis*) acclimatizes to a new ecosystem after introduction and which forage species it selects using the focal observation method.

The paper by T. Chigonda reviews the relationships between human livelihoods and biodiversity conservation in the rural areas of a semi-arid savanna and highlights the need for complete devolution of natural resource ownership and management to the grassroots levels in order to fully realise social and ecological sustainability.

The paper by G. Matseketsa et al. proposes a more socially and economically inclusive management approach, based on a stakeholder-driven access and benefit sharing framework, as essential in promoting a more positive form of reciprocity towards protected areas and nature conservation.

The paper by E. Mufandaedza et al. highlights variability in the influence of soil parameters (i.e., soil texture, soil pH, and available nitrogen (N) and phosphorus (P)) and level of tannins in tree barks in the distribution of mopane worms (*Imbrasia belina*) in savanna ecosystems.

The paper by C. Mutengu and W. Mhlanga analyses fish health and water quality and proposes the importance of long-term monitoring of freshwater ecosystems for sustainable fisheries management.

The paper by O. L. Kupika et al. illuminates the importance of local ecological knowledge held by rural communities adjacent to large protected areas on climate change- and ecosystem-based adaptation strategies and how this facilitates resilience in a changing environment.

